# Assessing the establishment risk for parthenogenetic populations of *Lissorhoptrus oryzophilus* in global rice-growing areas and potential economic impact in China

**DOI:** 10.3389/fpls.2024.1506418

**Published:** 2025-01-22

**Authors:** Luoyuan Li, Zhenan Jin, Ming Li, Yantao Xue, Jianyang Guo, Dong Jia, Ruiyan Ma, Zhichuang Lü, Xiaoqing Xian, Wanxue Liu

**Affiliations:** ^1^ College of Plant Protection of Shanxi Agricultural University, Jinzhong, China; ^2^ State Key Laboratory for Biology of Plant Diseases and Insect Pests, Institute of Plant Protection, Chinese Academy of Agricultural Sciences, Beijing, China

**Keywords:** invasive alien pest, *Lissorhoptrus oryzophilus* parthenogenetic populations, suitable area, ecological niche, potential economic loss

## Abstract

The rice water weevil, *Lissorhoptrus oryzophilus* Kuschel (Coleoptera: Curculionidae), threatens global rice production, with invasion events driven by its parthenogenetic populations. However, the global establishment risk in global rice-growing areas and potential economic losses and control benefits of the populations in invaded areas remain unclear. We applied an optimized MaxEnt model to predict the global suitable areas of the populations under current and future climate scenarios. Furthermore, we used @Risk software to estimate the potential economic losses and controlling benefit of this populations to rice production in China. Compared to its native range (North America), this populations has explored novel climates ecological niches in invaded areas (Europe and Asia) and occupies the broadest range of climatic ecological niches in Asia. The highly suitable area is primarily covered in rice-growing areas in China, the Korean Peninsula, and Japan, with all major rice-growing areas concentrated in these countries and regions. Under SSP1-2.6 and SSP5-8.5 emission scenarios, sum of suitable area for the populations in global rice-growing regions is projected to decrease by 0.35% and 0.26%, respectively, by the 2030s and 2050s. Moreover, @Risk analysis indicates that without control measures, the populations could cost China’s rice industry $18.95 billion, but management efforts could recover $17.54 billion. These results provide in-depth reference about the impact of climatic changes on the potential global suitable range of *L. oryzophilus* parthenogenetic populations and its economic impact on the rice industry in China.

## 1 Introduction

The advent of globalization and the concomitant enhancement of connectivity through international trade and the frequent movement of agricultural commodities has led to an increase in the incidence of invasive alien pests (IAPs) (Vilà et al., 2011). These present a substantial economic risk to worldwide agricultural crops, causing considerable losses and having a detrimental impact on human livelihoods (Cardinale et al., 2006; Daehler, 2003). A multitude of IAPs have undergone rapid evolutionary changes within their novel environments, driven by a combination of factors including genotype sorting, genetic recombination, interspecies hybridization, and natural selection. These changes have also occurred in response to the unique climatic conditions that they have encountered in their new habitats. (Guisan et al., 2014; Sexton et al., 2017). Notably, the IAPs with parthenogenetic reproduction have accelerated their spread and establishment in new environments due to their high adaptability and low energy consumption in reproduction (Graziosi and Rieske, 2014). Additionally, the impacts of climate change will lead to the higher rates, severity, damage, voltinism and geographic spread of IAPs to profound impact on agriculture production (Harrington et al., 2007; Hill et al., 2016; Ramasamy et al., 2022). To measure this threat and create effective biosecurity policies, it is essential to comprehend the origins of IAPs, their chances of establishing themselves in novel region, the region of establishing under climate change, and their possible economic impact in invaded areas. Therefore, assessing the distribution of globally suitable areas for IAPs under climate change, as well as estimating potential economic losses to staple crops in invaded areas and the benefits of control measures, is crucial for formulating pest management strategies to safeguard global food security (Ma and Ma, 2022).

*Lissorhoptrus oryzophilus* Kuschel (Coleoptera: Curculionidae) is native to the southern and eastern United States (Aghaee and Godfrey, 2014). In the southern region of Texas, bisexual and parthenogenetic populations of *L. oryzophilus* are present, but in California, only the parthenogenetic populations has been found to posed a huge threat to local rice production (Jiang et al., 2008; Aghaee and Godfrey, 2015). The adult weevils feed on rice leaves and lay their eggs after the fields are flooded, while the larvae feed on the roots, leading to poor root development, seeds dislodgement and reduced grain yields (Grigarick and Beards, 1965; Zou et al., 2004). *L. oryzophilus* is renowned for its highly invasive nature due to its ability to spread through a variety of mechanisms, including airborne dispersal, flight, swimming, and human-mediated transport. Additionally, it exhibits a great poliphagy (consuming a wide range of wild grasses) and adapts through various overwintering strategies (utilizing substrates like bunchgrass and leaf litter) (Chen et al., 2005; Saito et al., 2005; Adams et al., 2015). It is also important to recognize that a single individual is theoretically enough to initiate a new infestation due to its parthenogenetic reproduction in invaded areas (Saito et al., 2005). In the latter half of the 20th century, its parthenogenetic populations became world populations and invaded several high-yielding rice cultivation areas worldwide, including Europe (Italy), East Asia (Japan, China, South Korea and North Korea) and South Asia (India), causing significant economic losses in local agricultural production (Huang et al., 2017; Lupi et al., 2015; Saito et al., 2005; Ferrero, 2005; Giantsis et al., 2017; Lupi et al., 2010). These invasion events demonstrate that the *L. oryzophilus* females have a strong adaptive capability to new climatic environments (Saito et al., 2005; Caldara et al., 2004; Lupi et al., 2015). Previous studies have used CLIMEX to predict the suitable areas for this populations in China based on its biological characteristics and environmental adaptations (Mao et al., 1997). Qi et al. (2012) used MaxEnt to explored the suitable areas of the populations in rice-growing areas in China. Xie et al. (2022) predicted the suitable areas of the populations under current and future climatic conditions by MaxEnt. However, as a widely prevalent invasive populations globally, the establishment risk of this populations in global rice cultivation areas remain unknown, and there have been no quantitative assessments reported regarding the regional potential economic losses and control benefits in invaded areas, especially in China where there is high dependence on rice supply (Bin Rahman and Zhang, 2023). It is essential to predict the suitable areas of *L. oryzophilus* parthenogenetic populations in global rice growing areas under climate change conditions so that targeted management and control measures to mitigate increasing losses.

Species distribution models (SDMs) use environmental variables from known species locations to predict their distributions and ecological needs; these models are commonly applied in invasion biology, conservation biology, global change biology, and pathogen risk assessments (Raffini et al., 2020; Pili et al., 2020; Larson and Olden, 2012). The MaxEnt model combines machine learning with the principle of maximum entropy, and it is widely used in invasive species risk assessment due to its flexibility and ease of interpretation (Phillips and Dudík, 2008; Elith et al., 2011; Jin et al., 2022; Yang et al., 2022). The ecological niche shifts of IAPs in invaded habitats have been extensively documented (Sexton et al., 2017; Guisan et al., 2014). Understanding the ecological niche characteristics occupied by invasive species in invaded areas also provides new insights into their impacts. The n-dimensional hypervolume framework has been used to build and compare ecological niche characteristics, which are more comprehensive than two-dimensional methods that condense multiple niche variables into principal components (Blonder et al., 2018, 2014). Multidimensional methods provide a fuller depiction of niche differences by capturing all variability across niche dimensions (Pack et al., 2022; Pili et al., 2020). Additionally, we are interested in exploring changes in niche characteristics of specific variables related to the environmental suitability of *L. oryzophilus* parthenogenetic populations. This is feasible within the n-dimensional framework, as centroids for each niche dimension can be computed, further quantifying how species differ across various environmental factors during the invasion process. Typically, predictions of potential economic losses caused by harmful organisms rely on @RISK software, which utilizes Monte Carlo stochastic simulation methods. By constructing models of potential economic losses under different scenarios and simulating outcomes using various probability distributions, @RISK software can estimate the potential economic impact of harmful organisms (Li and Qin, 2018). Previous studies have used @RISK software to predict the potential economic losses and control costs caused by *Bactrocera cucurbitae* and *Spodoptera frugiperda* on host plants in China (Sun et al., 2018; Qin et al., 2020). Given that China is the largest rice producer globally (https://www.fao.org/), this study aims to utilize the @RISK stochastic model to assess the potential economic impact of *L. oryzophilus* parthenogenetic populations on China’s rice industry.

Despite extensive efforts both domestically and internationally in developing quantitative risk assessment models and software for IAP, existing models and software to date cannot independently achieve comprehensive assessments covering the invasion potential, suitable areas, and potential economic losses from IAPs (Li and Qin, 2018). Therefore, it is necessary to integrate these models and software organically in IAPs risk analysis framework (Li and Qin, 2018). We construct a new framework for global establishment risk assessment of *L. oryzophilus* parthenogenetic populations (**Supplementary Figure S1**). Specifically, (1) reconstruct the invasion history of the populations across recorded countries; (2) identify significant environmental variables affecting the suitable areas of the populations; (3) predict the suitable areas of the populations in global rice-growing areas under two climate change conditions; (4) construct ecological niche characterizations for North America, Europe, and Asia using four major bioclimatic factors that largely impact model results to characterize ecological differences in suitable habitats for the populations. (5) evaluate the potential economic losses of the populations to rice industry under unmanaged scenario and managed scenario.

## 2 Materials and methods

### 2.1 Species distribution record

**Figure 1:**
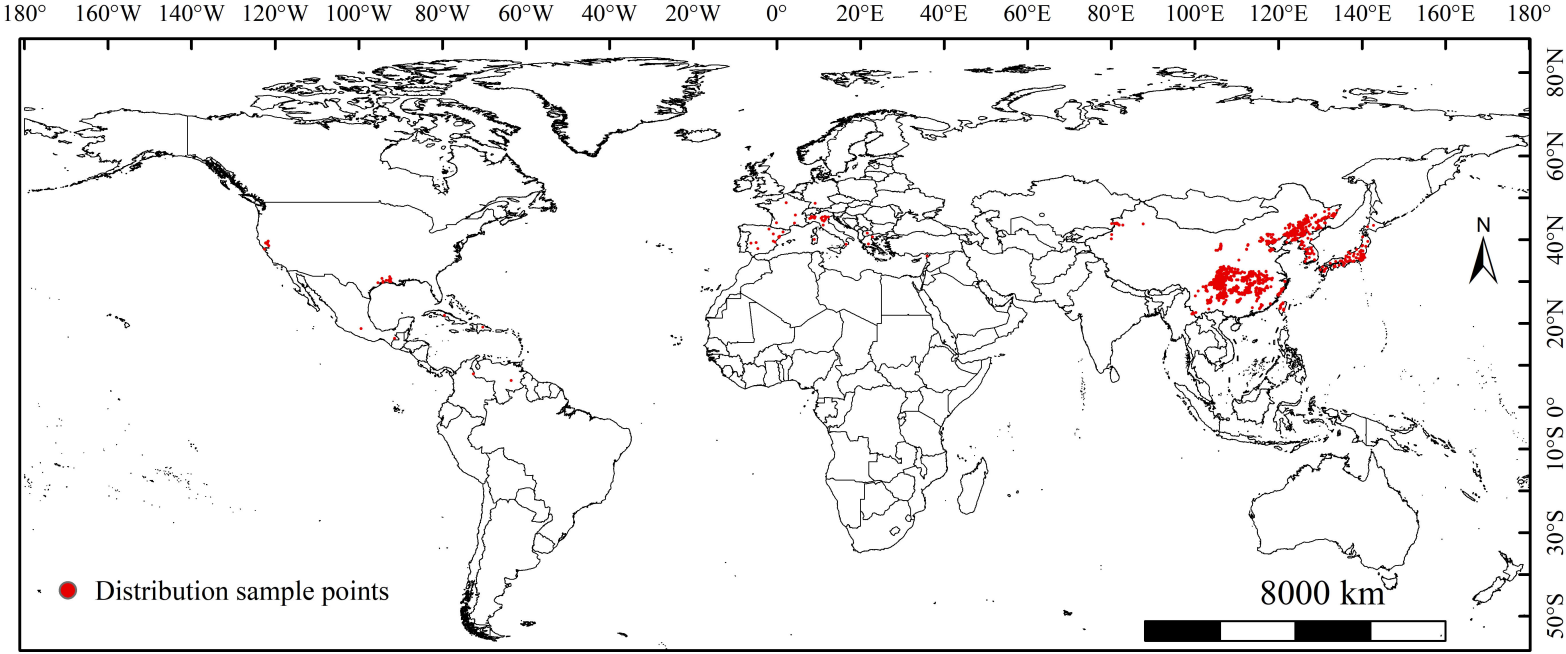
Global occurrence sampling points of *Lissorhoptrus oryzophilus* parthenogenetic populations.

We obtained global occurrence records of *L. oryzophilus* parthenogenetic populations from the Global Biodiversity Information Facility (https://www.gbif.org/; accessed on February 7, 2023 https://doi.org/10.15468/dl.tnzme5), Bold Systems v4 (http://www.boldsystems.org/; accessed on February 21, 2023) and published literature (China National Knowledge Infrastructure: https://www.cnki.net; Web of Science, https://www.webofscience.com/; accessed on May 9, 2023). A total of 1991 global occurrence records for *L. oryzophilus* parthenogenetic populations were collected. Most of the sampling points are concentrated in central and northeastern China, as well as mainland Japan. Only a few samples have been recorded in the eastern and southern United States, and southern Europe. To avoid spatial redundancy of the sample points, we also used ENMTools to eliminate spatial autocorrelation by screening occurrence records, ensuring each raster retained a single occurrence record (Warren et al., 2010). Finally, 1500 occurrence records of *L. oryzophilus* parthenogenetic populations were retained for constructing the MaxEnt model (**Figure 1**).

We reconstruct parthenogenetic and bisexual populations of *L. oryzophilus* invasion history in native (the United States) and invasive range based on the time of the first reported in EPPO Global Database (https://www.eppo.int/; accessed on February 21, 2023) and the literature for different invasive region (Web of Science, https://www.webofscience.com/; accessed on May 9, 2023) (**Supplementary Table S1**). For the first occurrence records of *L. oryzophilus* in different provinces of China, we conducted a detailed search based on CNKI (China National Knowledge Infrastructure: https://www.cnki.net) and the National Agricultural Plant Quarantine Pests Distribution Administrative Region Directory (www.moa.gov.cn) (**Supplementary Table S1**). All the countries, provinces and states with documented first reported are visualized using different colors in ArcGIS 10.8 software.

### 2.2 Environmental variables

For climatic parameters, we obtained 19 bioclimatic factors (**Supplementary Table S2**) and altitude variables from the World Climate Database (http://www.worldclim.org/; accessed on October 3, 2022) at a resolution of 5 arc-min (10.0 km resolution at the equator). These 19 bioclimatic factors were derived from minimum temperature, maximum temperature, and precipitation data from 1970 to 2000 to create suitability maps under current climate conditions. We also incorporated the HII (Human Influence Index (https://beta.sedac.ciesin.columbia.edu/data/set/wildareas-v2-human-influence-index-geographic)) into our model. To avoid model overfitting, we used the ENMTools to examine correlations among 21 predictor variables and remove highly correlated environmental factors (| *r* |< 0.8, Pearson) to reduce the collinearity between predictor variables (**Supplementary Figure S2**) (Graham, 2003; Favretti, 2018). Finally, the ten predictor variables were chosen based on their importance for the MaxEnt model: mean diurnal range (Bio 2), temperature seasonality (Bio 4), mean temperature of warmest Quarter (Bio10), precipitation of driest month (Bio 14), precipitation seasonality (Bio 15), precipitation of wettest quarter (Bio 16), precipitation of warmest quarter (Bio 18), precipitation of coldest quarter (Bio 19), altitude (Alt) and Human Influence Index (HII) (**Supplementary Table S3**).

We also obtained bioclimatic variables were from the Beijing Climate Center Climate System Model (BCC-CSM2-MR) (http://www.worldclim.org/; accessed on October 3, 2022), developed by the National Climate Center based on the CMIP6 of the sixth assessment report (AR6) of the Intergovernmental Panel on Climate Change (IPCC). Compared to BCC-CSM1.1m, the BCC-CSM2-MR shows notable advancements in tropospheric temperature and circulation on global and East Asian scales, as well as in climate variability across different time scales (Wu et al., 2019). In our study, SSP1-2.6 (low-emission scenario) and SSP5-8.5 (high-emission scenario) in the 2030s (average of 2041–2060) and 2050s (average of 2041–2060) were included to predict the suitable areas under future climatic conditions.

### 2.3 Model optimization and parameterization

The default parameter settings in MaxEnt can lead to models that are either overly complex or too simplistic (Warren and Seifert, 2011). Therefore, the alternative combinations of feature classes (FCs) and regularization multipliers (RMs) optimization of the MaxEnt model was conducted using the ENMeval package in the R software to obtain appropriate parameter settings (Morales et al., 2017; Muscarella et al., 2014). The MaxEnt model has five basic features: linear (L), quadratic (Q), hinge (H), product (P), and threshold (T) (Santana et al., 2019). We set five parameter combinations for these five arithmetic features as L, LQ, H, LQH, and LQHP (Muscarella et al., 2014). The regularization multipliers were set from 0.5 to 6, with a 0.5 interval, resulting in 60 combinations of model parameters.

We selected the minimum values of delta Akaike information criterion correction (ΔAICc) as criteria (Jarnevich et al., 2015). A model with a lower AICc score indicates lower complexity and better fit, making it the preferred choice. Thus, select the model with the minimum ΔAICc value (choose ΔAICc = 0) to build the final MaxEnt model. The FC parameters were set as LQHP with a regularization multiplier of 0.5. The global occurrence records of *L. oryzophilus* were divided, and 75% and 25% were selected as the training and testing datasets, respectively (Merow et al., 2013; Dudík et al., 2005). We also set the maximum iteration value to 500, selected the run type as “Bootstrap”, the maximum background points were 10,000, 10 times running repetition of the model and random seeds were selected to enhance model randomness (Warren and Seifert, 2011). The other parameters were maintained at their default settings.

### 2.4 Model result evaluation and GIS analysis

We used the AUC of the receiver operating characteristic (ROC) analysis to assess and calibrate the accuracy of the MaxEnt model. The AUC was examined for additional accuracy analyses, including the agreement between introduced observations and predictive logistic regression results (Elith et al., 2011; Ficetola et al., 2007). AUC values range from 0 to 1, with larger values indicating greater credibility in model performance: the values< 0.6, 0.6–0.9, and > 0.9 were considered poor, useful, and excellent, respectively (Liu et al., 2005). The probabilistic prediction results generated by the MaxEnt model are presented in a grid raster format, with values ranging from 0 to 1. Using the spatial projection outputs from the MaxEnt model, we estimated the future suitable areas of the species under climate change scenarios (SSP1-2.6 and SSP5-8.5). The suitable areas were divided into suitable habitats and unsuitable habitats based on the cloglog threshold value of the maximum training presence (MTP). The cloglog threshold for the MTP was set at 0.1039, classifying areas below this threshold as unsuitable habitats and those above as suitable (Liu et al., 2005). Accordingly, the suitable areas of the pest were reclassified into four categories: unsuitable habitats (0-MTP), low suitable habitats (MTP-0.4), moderate suitable habitats (0.4-0.6), and high suitable habitats (0.6-1).

The successful invasion and establishment of new areas by IAPs also depend on the suitability of the preferred host (de Godoy et al., 2023; Huber and Geist, 2019; Dukes and Mooney, 1999). To assess the impact of *L. oryzophilus* parthenogenetic populations on global rice- growing areas, we obtained distribution data in global rice-growing areas from the EarthStat database (**Supplementary Figure S3**; www.earthstat.com). The global rice-growing area was overlaid on the suitable areas of the populations under current and future climate scenarios. Additionally, we evaluated the proportion of the range of suitable areas for the species in the global rice-growing area to quantify the impact of this species parthenogenetic populations on global rice cultivation areas under current and future climate scenarios.

### 2.5 Hypervolume of climate ecological niche

In this study, we used four predictor variables (Bio18, Bio4, Bio10 & Bio16), which significantly contribute to the model results, to constructed ecological niche characterization of *L. oryzophilus* parthenogenetic populations for North America, Europe, and Asia. The analysis data comes from extracting values from four predictor variables using known occurrence points in ArcGIS 10.8. For each continent, we used a full set of occurrence records to construct a complete ecological niche hypervolume. We used the “hypervolume” package to analysis in R software (Blonder et al., 2018). Based on the selected four climate variables, we used the Z-score to standardize the four predictor variables for constructing three niche hypervolume structures in three continent and adopted a cross-validation approach to select the bandwidth of each variable axis for optimization (Blonder et al., 2018; Jin et al., 2024). Then we mapped hypervolumes onto orthogonal axes comprising pairs of climatic variables and characterized the ecological niche similarity of *L. oryzophilus* across the three continents by calculating hypervolume distances between centers of mass (Euclidean distances) and similarities between hypervolume structures (Sorensen indices) (Mammola, 2019).

### 2.6 Assessment of economic losses to rice production in China under current climate scenarios

We analyzed and assesses the potential economic losses to rice production in China caused by *L. oryzophilus* parthenogenetic populations under unmanaged scenario and managed scenario (Ullah et al., 2023; Xu et al., 2020):

Unmanaged scenario:

Potential economic loss caused by production quantity decline-F1


F1=Q∗I∗R∗Pa/(1−I∗R)

Managed scenario:

Management costs-F2


F2=S∗I∗C

Potential economic loss after management-F3


F3=Q∗I∗E∗Pa/(1−I∗E)

Potential economic loss under management scenario-F4


F4=F2+F3

Potential savings after management-F5


F5=F1−F4

We obtained the statistical data on rice production and planting area for each province from 2018 to 2022 from the National Bureau of Statistics (http://www.stats.gov.cn/) and Strategic Reserves Administration (http://www.chinagrain.gov.cn/). Based on the market price of pesticides in China and the suitable area of the populations, we assessed the required investment in prevention cost (Xu et al., 2020; He et al., 2016). We combined the rice production and planting area data with the proportion of suitable areas for the populations in each province to calculate the annual rice production and planting area within its suitable areas (Qin et al., 2020). The specific indicator setting for harm and economic parameters under Unmanaged and managed scenarios are shown in **Table 1**. All parameters were entered into the @Risk software as distribution functions (using the Pert function for fitting), and a Latin hypercube sampling method was employed to draw samples from each distribution with a total of 100,000 iterations conducted (Ullah et al., 2023).

**Table 1:** Calculate specific parameter settings for potential economic losses.

Scenarios	Parameters types	Unite	Parameters setting	Reference
Unmanagement scenario	The yield of rice plants in suitable habitats - Q	t	Pert (192633999.68, 194501221, 196083353.35)	http://www.stats.gov.cn/
	The damage rate of rice-I	%	Pert (10, 30, 50)	(Zou et al., 2004)
	The yield loss rate of rice-R	%	Pert (15.9, 51.35, 86.8)	(Zhou et al., 2007)
	Rice market price-Pa	$/t	Pert (374.12, 402.32, 544.83)	http://www.chinagrain.gov.cn/
management scenario	The area of rice plants in suitable habitats -S	hm^2^	Pert (27363597.06, 27683966.83, 27966379.44)	http://www.stats.gov.cn/
	Unit prevention cost-C	$/t	Pert (5.87, 39.85, 110.64)	(He et al., 2016)
	Unit yield of rice in suitable area-W	t/hm^2^	Pert (6.99, 7.03, 7.07)	–
	Control effect -M	%	Pert (72.44, 78.35, 84.25)	(He et al., 2016)
	Benefit correction coefficient -D	–	2	(Xu et al., 2020)
	Economic loss level -E	%	E=C/(W*Pa*M) *D*100%	–

## 3 Results

### 3.1 Invasion history of *L. oryzophilus*

**Figure 2:**
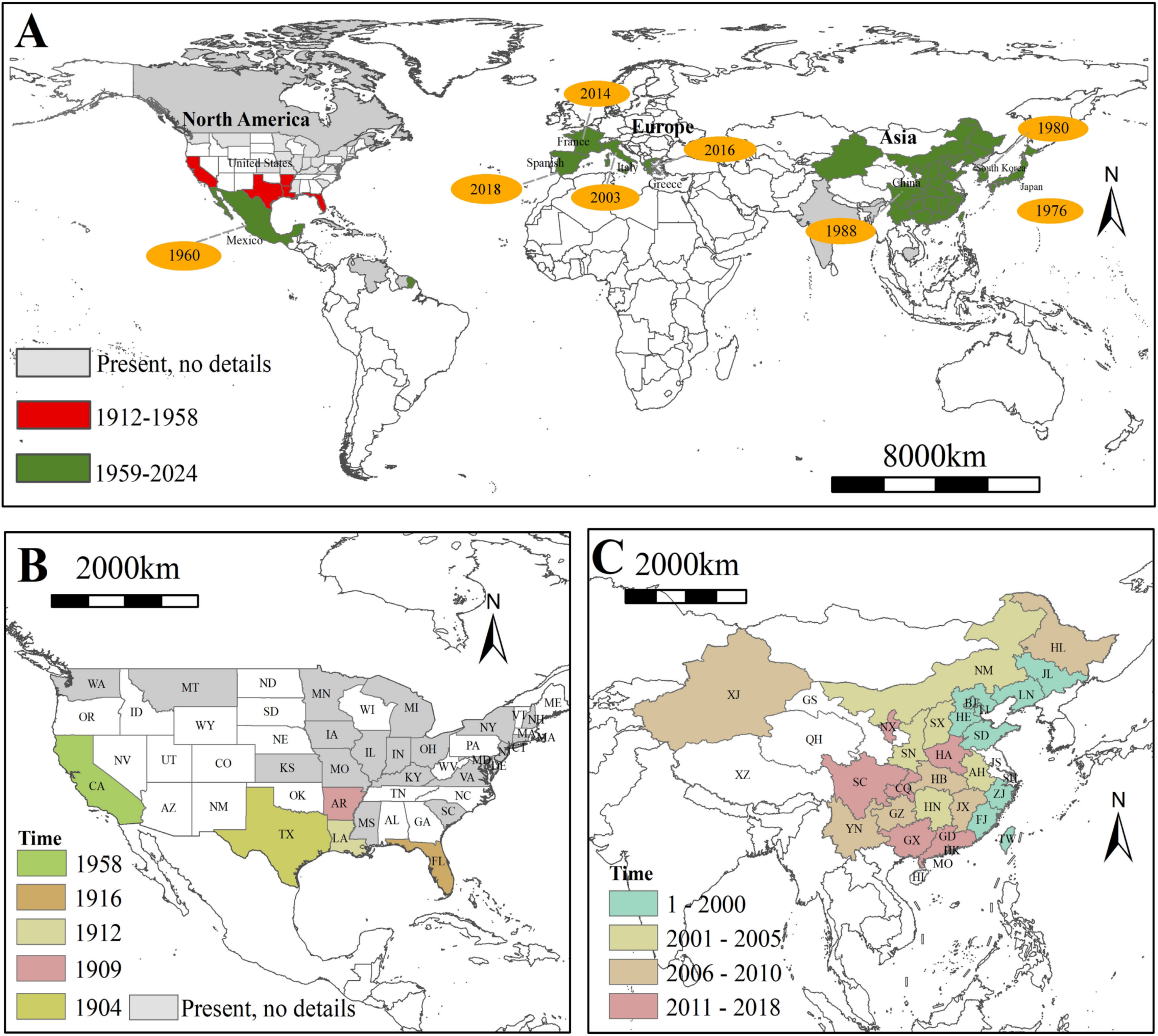
**(A)** Global invasion history reconstruction of *Lissorhoptrus oryzophilus*; **(B)** Historical spread dynamics of *L. oryzophilus* in the United States; **(C)** Historical spread dynamics of *L. oryzophilus* in China.

Since the first discovery the bisexual populations of this species in Texas in 1904, subsequent findings of the bisexual populations occurred within 20 years in Louisiana, Kansas, and Florida (**Figures 2A, B**). Following the initial report of the parthenogenetic populations in California in 1958, this populations rapidly invaded northern Latin America, eastern Asia, and southern Europe over the next 70 years, propelled by the acceleration of global trade (**Figure 2A**). As of 2020, the distribution of this species is primarily concentrated in the Northern Hemisphere, particularly in regions with extensive rice cultivation, such as China and Japan (**Figure 2**). Especially in China, since the first discovery of the parthenogenetic populations of *L. oryzophilus* in Hebei Province in 1988, this populations rapidly spread along the coastal regions of China during the 1990s. After entering the 21st century, the populations gradually showed a trend of spreading from the coastal areas to the inland regions. As of 2024, approximately 5/6 of the provinces have recorded the distribution of the parthenogenetic populations in China (**Figure 2C**).

### 3.2 Model accuracy evaluation and importance of bioclimatic variables

Based on the model optimization results, with RM = 0.5 and FC = LQHP, the ΔAICc value was 0. Therefore, these were selected as the final parameter settings (**Supplementary Figure S4A**). The resulting mean training and testing AUC values were 0.939 (**Supplementary Figure S4B**), indicating the outcomes of the model were reliable. The precipitation of the warmest quarter (Bio18, 57.9%), temperature seasonality (Bio4, 22%), mean temperature of the warmest quarter (Bio10, 13.7%), and precipitation of the wettest quarter (Bio16, 1.7%) were the four most significant bioclimatic variables affecting the suitable areas of parthenogenetic populations (**Supplementary Table S3**). The effect of precipitation on the suitable areas of the populations was more significant than that of temperature, as indicated through the jackknife and percent contributions (**Supplementary Table S1**, **Supplementary Figure S5**). According to the precipitation during the warmest quarter (Bio18), the probability of survivability increased from 0.03 to 0.86 within the range of 26.50 to 562.20 mm and subsequently declined rapidly (**Supplementary Figure S6A**). The response curves of temperature seasonality (Bio4) showed that within the range of 1.86–751.26, the probability of occurrence increased from 0.006 to 0.92, rapidly decreased until approximately 976.63, the probability of occurrence increased from 0.92 to 0.38 (**Supplementary Figure S6B**). Regarding the Bio10 and Bio16 precipitation variables, the probability of occurrence peaked when the variables were 26.36°C and 1567.56 mm, respectively (**Supplementary Figures S6C, D**).

### 3.3 Suitable areas of *L. oryzophilus* parthenogenetic populations under the current climate

**Figure 3:**
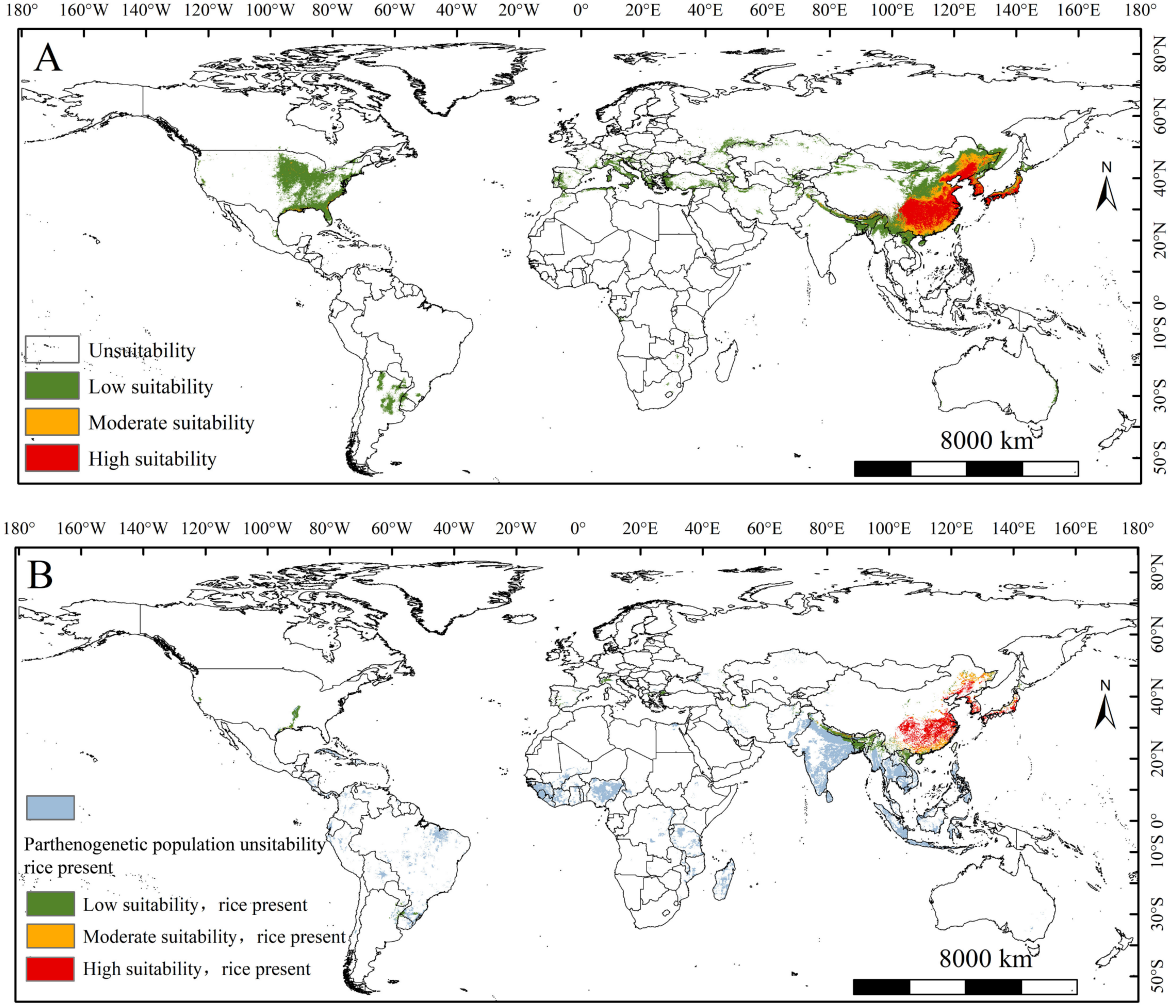
**(A)** The suitable areas of *Lissorhoptrus oryzophilus* parthenogenetic populations under current climatic scenarios. **(B)** The suitable areas of *L. oryzophilus* parthenogenetic populations in global rice-growing areas under current climatic scenarios.

Current global suitable areas forecast of the populations are depicted in **Figure 3**. The global suitable areas for the parthenogenetic populations establishment was 1068.4 × 10^4^ km^2^ under the current climate (**Table 2**). Highly suitable habitats for the populations are predicted in the northeastern and southern of China, the western Japan, and Korea peninsula (**Figure 3A**). The area of high suitability was 143.83 × 10^4^ km^2^ (**Table 2**). Moderately suitable habitats for the populations are predicted in the northeastern and southern of China (**Figure 3A**). The area of moderate suitability was 209.67 × 10^4^ km^2^ (**Table 1**). Lowly suitable habitats for the populations are predicted in eastern and southeastern United States, northern Argentina, southern Europe (Italy, Macedonia, Greece, France, Spain), western Asia (Turkey and Kazakhstan), northern Southeast Asia (Vietnam, Myanmar, Laos and northeastern India) and the northeastern and southern of China (**Figure 3A**). The area of low suitability was 714 × 10^4^ km^2^ (**Table 2**).

**Table 2:** Predicted suitable habitat areas for *Lissorhoptrus oryzophilus* parthenogenetic populations under current and future climatic scenarios (×10^4^km^2^).

	Current	SSP1-2.6			SSP5-8.5		
		2030s	2050s	Ave	2030s	2050s	Ave
low suitability	714.90	772.86 (8.11)	659.29 (-7.78)	716.08 (0.16)	723.79 (1.24)	807.62 (12.97)	765.71 (7.11)
moderate suitability	143.83	149.69 (4.07)	152.65 (6.13)	151.17 (5.10)	152.42 (5.97)	145.38 (1.08)	148.90 (3.52)
high suitability	209.67	196.99 (-6.05)	206.90 (-1.32)	201.95 (-3.68)	198.71 (-5.23)	196.20 (-6.42)	197.46 (-5.83)
Total suitability	1068.40	1119.54 (4.79)	1018.85 (-4.64)	1069.20 (0.07)	1074.93 (0.61)	1149.20 (7.56)	1112.07 (4.09)
Total suitability in global rice-growing areas	221.94	225.10 (1.43)	217.22 (-2.13)	221.16 (-0.35)	223.34 (0.63)	219.368 (-1.16)	221.35 (-0.26)

The parentheses following the numbers indicate the percentage change in suitable habitat area relative to the current climate scenario across different time periods and climate scenarios.

The range of suitable areas for the populations within the global rice-growing area is 221.94 × 10^4^ km^2^, accounting for 30.6% of the total global rice-growing area under current conditions (**Figure 3B**, **Table 2**). The regions primarily affected by this populations in the rice industry include North America (the southern unite states), South America (Southern Brazil), southern Europe (Northern Italy and coastal areas of Spain), and Asia (northern Vietnam, northern Myanmar, northeastern and northern India, northeastern and southern of China, Western Korean Peninsula, Southern and Western Japan). Among these regions, the populations have notably impacted rice-growing areas in China, the Korean Peninsula, and Japan, with all major rice-growing areas concentrated in these countries and regions (**Figure 3B**).

### 3.4 Suitable areas of *L. oryzophilus* parthenogenetic populations under future climate scenarios

**Figure 4:**
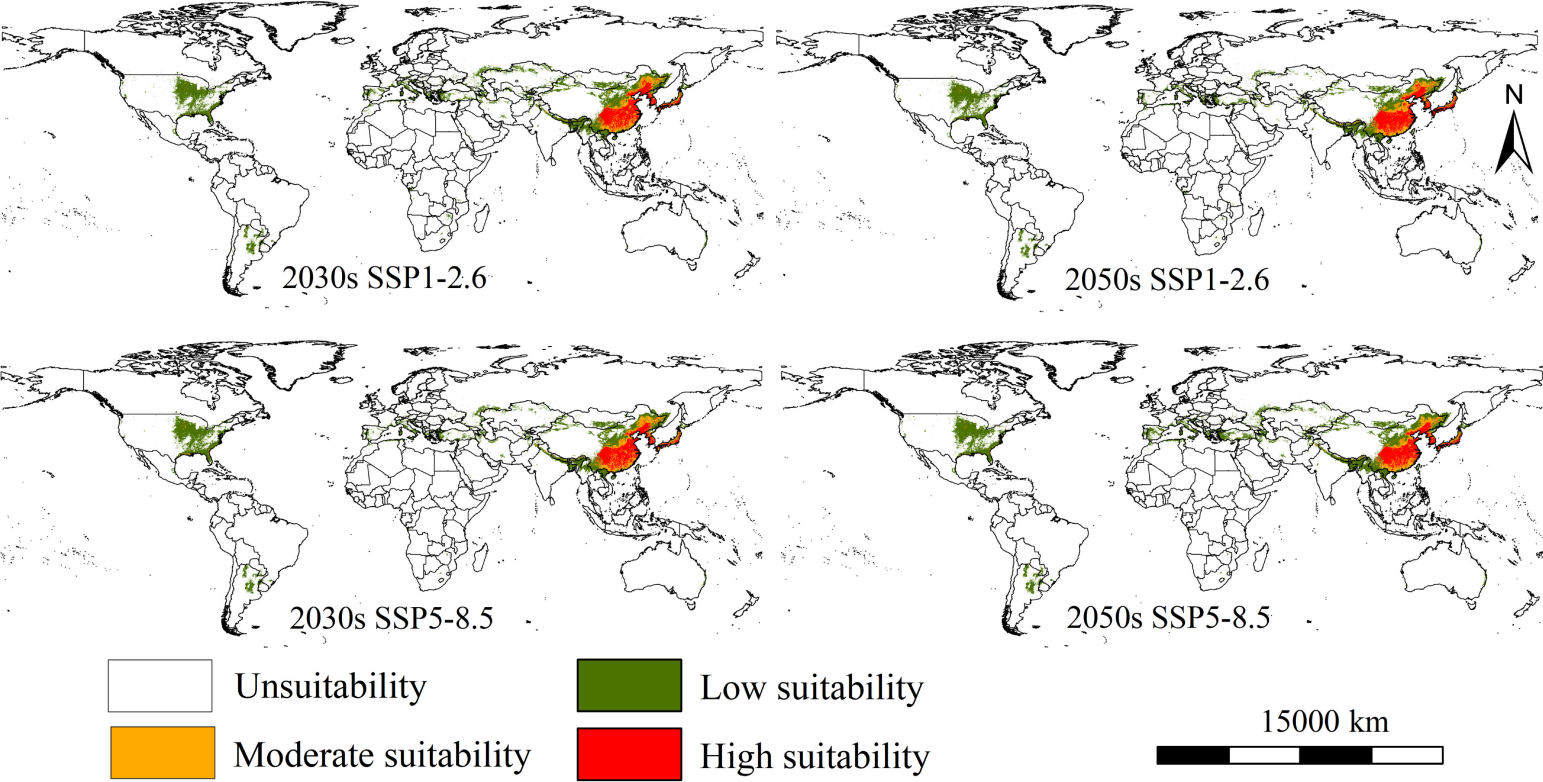
The suitable areas of *Lissorhoptrus oryzophilus* parthenogenetic populations under two future climatic scenarios in 2030s and 2050s (SSP1-2.6 (low-emission scenario) and SSP5-8.5 (high-emission scenario)).

The suitable areas of the parthenogenetic populations under two climate change scenarios (SSP1-2.6 and SSP5-8.5) and in two time periods (2030s and 2050s) is shown in **Figure 4**. Overall, compared to current climate scenarios, the populations present insignificant changes in global suitability under two future climate scenarios. Its primary suitability remains concentrated in eastern North America, southern South America, southern Europe, and eastern Asia. Northeastern China, western Korean Peninsula, and western and southern parts of Japan continue to be identified as major high suitability areas (**Figure 4**). The low-emission SSP1-2.6 scenario suggested the total suitable habitat areas are 1069.20 × 10^4^ km^2^, with an increase of 0.07% in the current suitable area for the establishment of the populations (**Table 2**). The low and moderate suitability areas cover 716.07 × 10^4^ km^2^ and 151.17 × 10^4^ km^2^ (increase 0.16% and 5.10% compared to the current area), while high suitability areas are 201.95 × 10^4^ km^2^ (decrease 3.63% compared to the current area) (**Table 2**). The MaxEnt model further predicted higher habitat suitability areas and spread of RSW under high-emission scenario SSP5-8.5 in 2030s and 2050s. The high-emission SSP5-8.5 scenario suggested the total suitable habitat areas are 1111.80 × 10^4^ km^2^, with an increase of 4.0% in the current suitable area for the establishment of the populations (**Table 2**). The low and moderate suitability areas cover 765.71 × 10^4^ km^2^ and 151.17 × 10^4^ km^2^ (increase 7.1% and 3.5% compared to the current area), while high suitability areas are 201.95 × 10^4^ km^2^ (decrease 5.82% compared to the current area) (**Table 2**).

**Figure 5:**
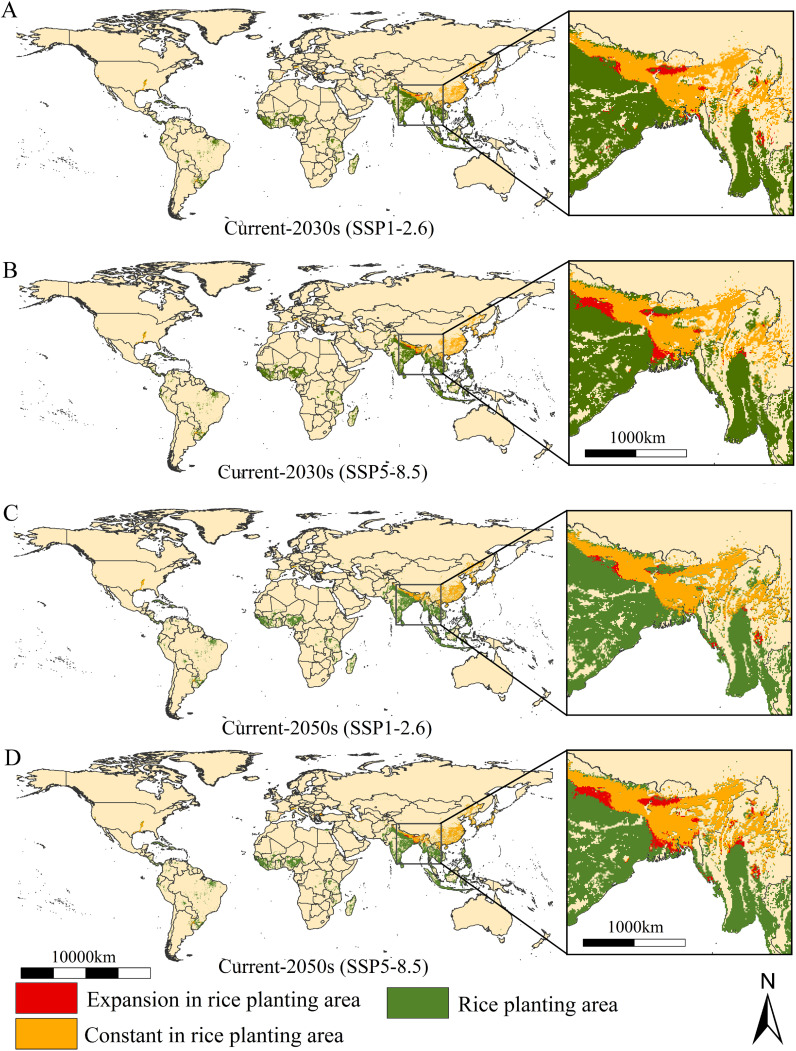
The change of the suitable areas of *Lissorhoptrus oryzophilus* parthenogenetic populations in global rice growing areas under future climatic scenarios from current to 2030s **(A, B)** and 2050s **(C, D)** under SSP1-2.6 (low-emission scenario) and SSP5-8.5 (high-emission scenario).

Changes in the establishment risk of the populations in global rice-growing areas under two future climate scenarios is shown in **Figure 5**. Overall, sum of suitability areas will decrease to 221.16×10^4^ km^2^ and 221.35×10^4^ km^2^ (decrease 0.35% and 0.26% compared to the current area) (**Table 2**). The establishment risk of this populations in global rice-growing areas is primarily concentrated in North America (the southern unite states), South America (Southern Brazil), southern Europe (Northern Italy and coastal areas of Spain), and Asia (northern Vietnam, northern Myanmar, northeastern and northern India, northeastern and southern of China, Western Korean Peninsula, Southern and Western Japan) under SSP1-2.6 and SSP5-8.5 scenarios (**Figure 5**). Fortunately, the increased suitability areas for the establishment of the populations are generally stable, concentrated in northern and northeastern India, and northern and central Myanmar (**Figure 5**).

### 3.5 Ecological niche similarity of *L. oryzophilus* parthenogenetic populations in three continents

**Figure 6:**
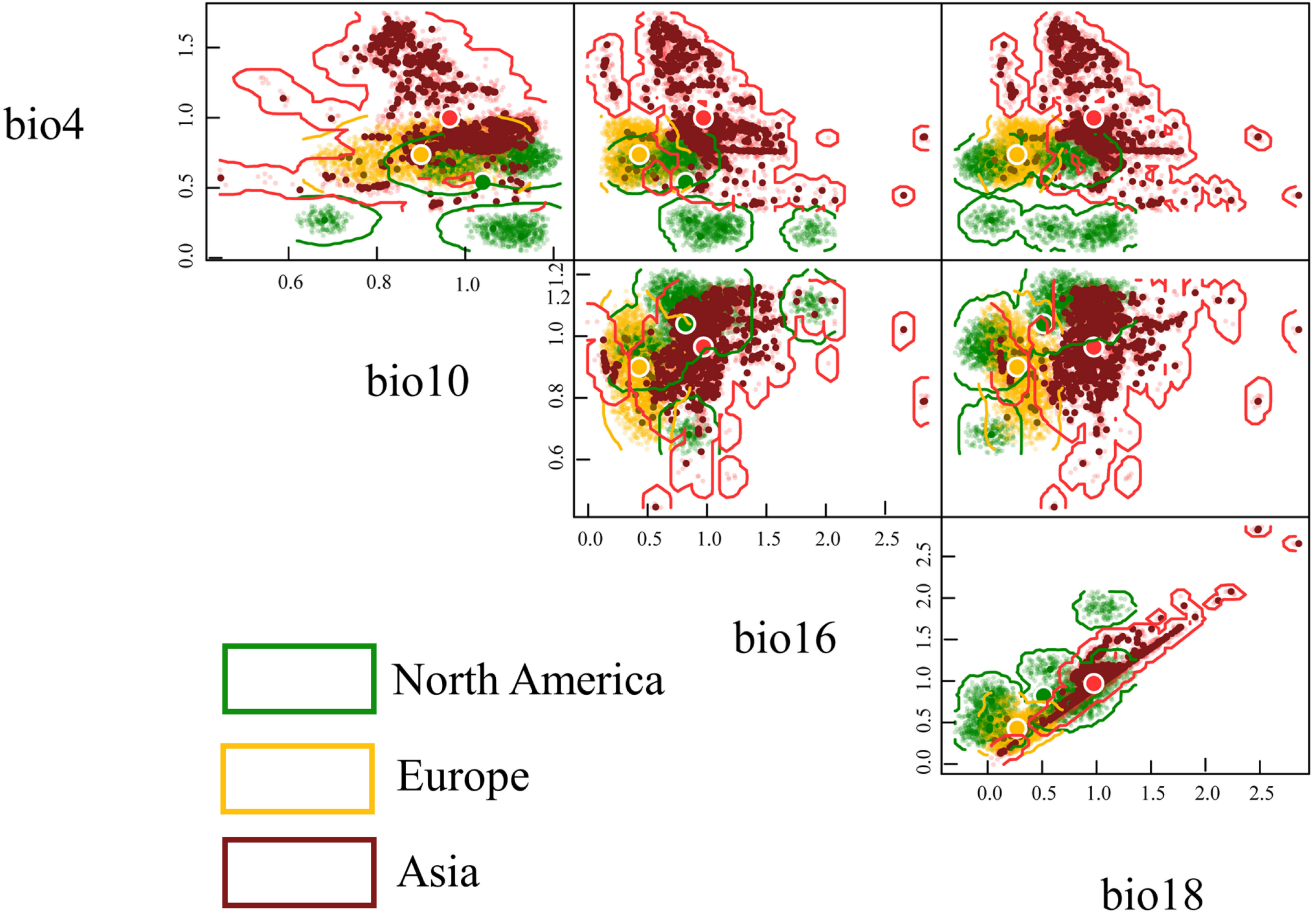
Based on the n-hypervolume and four key climatic variables to construct the climatic niche characteristics of *Lissorhoptrus oryzophilus* parthenogenetic populations across North America, Europe, and Asia (bio4 (Temperature Seasonality), bio10 (Mean Temperature of Warmest Quarter), bio16 (Precipitation of Wettest Quarter), bio18(Precipitation of Warmest Quarter)).

Here, we utilized the n-hypervolume framework to comprehensively assess the niche similarity between the native range (North America) and invasive ranges (Europe and Asia) using the Sorensen index and Euclidean distance (**Table 3**). Overall, this population exhibits significant differences in climatic niche occupancy between the invasive and native ranges. The Euclidean distance is 0.5495 between European and North American populations, with a Sorensen index of 0.1482. However, Asian population shows even greater Euclidean distance (0.6727) and a smaller Sorensen index (0.0133) relative to North American populations. Furthermore, compared to North America and Europe, this population occupies a larger and more distinct climatic niche space in Asia (**Figure 6**). Compared to its native range, this population occupies a climatic niche in Asia where, except for Bio10 (mean temperature of the warmest quarter) which has expanded towards lower levels, the other three variables (Bio4 (Temperature Seasonality), Bio16 (Precipitation of Wettest Quarter), Bio18 (Precipitation of Warmest Quarter)) have expanded towards higher levels (**Figure 6**).

**Table 3:** Climatic ecological niche similarity of *Lissorhoptrus oryzophilus* parthenogenetic populations in North America, Europe, and Asia (above the diagonal is the Sorensen index, below the diagonal is centroid distance).

	Euclidean distance\Sorensen		
	North America	Europe	Asia
North America		0.1482	0.0133
Europe	0.5495		0.0006
Asia	0.6727	0.9319	

### 3.6 The potential economic losses caused by *L. oryzophilus* parthenogenetic populations in China and their controlling benefit

Under the unmanaged scenario (F1), the potential economic losses of *L. oryzophilus* parthenogenetic populations on the rice industry were estimated to range from $5.44 billion to $32.46 billion USD at a 95% confidence level. Sensitivity analysis results showed that the ranking of the factors influencing these potential losses under unmanaged scenario include the rice yield loss rate (R), the damage rate of rice (I), the rice market price (Pa), and the yield of rice in suitable habitats (Q) (**Supplementary Figure S7A**).

Under the management scenario (F2), the potential economic loss of rice in China due to the populations range from $0.10 billion to $0.83 billion USD at a 95% confidence level, with a mean value of $0.47 billion USD. The potential economic loss from management costs (F3) ranges from $0.26 billion to $2.19 billion USD at a 95% confidence level, while the loss after management (F4) ranges from $0.36 billion to $3.02 billion USD, with mean values of $1.22 billion and $1.69 billion USD, respectively. Potential savings after management (F5) range from $4.41 billion to $30.68 billion USD at a 95% confidence level, with a mean value of $17.54 billion USD. Sensitivity analysis results showed that the ranking of the factors influencing these potential losses under the management scenario includes the loss rate after the unit prevention cost (C), the damage rate of rice by the populations (I), the area of rice plants in suitable habitats (S), the yield of rice in suitable habitats (Q), the rice market price (Pa), and the control effect of the populations (M) (**Supplementary Figure S7B**).

## 4 Discussion

Rice is the most important crop in the world because half of the global populations consume it daily. In some Asian countries, rice provides over 70% of the calorie supply (GRiSP, 2013). Therefore, rice is considered one of the most strategically significant commodities worldwide, closely intertwined with global food security, economic growth, employment, social stability, and regional peace (Mehrabi et al., 2018). However, the transport of pests beyond their native ranges by human actions is breaking down biogeographical barriers and threatening local rice production, and invasive alien pests also continually explore new climate characteristics during this process (Capinha et al., 2015; Vilà et al., 2011). Indirectly, negative impact of climate change on rice yield by altering the suitable areas range of pests, increasing the occurrence of pests and diseases and exacerbating natural disasters, thus posing a threat to food security (Tilman et al., 2011; Lori, 2021; Rezaei et al., 2023).

### 4.1 Humid and hot environments are favorable for the occurrence of the populations

Our results revealed that the effect of precipitation factors on the prevalence of *L. oryzophilus* parthenogenetic populations was significantly greater than that of temperature factors. Specifically, the precipitation of the warmest quarter (Bio18) and accounted for 57.9% and 13.7% of the contribution rate to the MaxEnt result and ranging from 685.19 to 753.33 mm for the precipitation of the warmest quarter were considered high suitability for the populations. Previous studies showed that the populations oviposits in the submerged portion of the rice seedlings in summer and the peak oviposition typically coincides with the establishment of a flood, fostering accelerated larval growth rates under these conditions (Bowling, 1972; Tindall et al., 2013; Stout et al., 2002). Previous studies indicate that most 1st-generation adults end diapause after exposure to 10°C, 15°C, and 20°C, while diapause intensity is higher at 25°C, possibly to avoid extreme high temperatures after emergence (Jiang et al., 2004). However, the 2nd-generation adults are less likely to face high temperatures, resulting in lower diapause intensity (Jiang et al., 2004). In our result, the mean temperature the warmest quarter (Bio10) maintained between 10°C and 33°C, the probability of presence for this populations will exceed 0. The previous results corroborate our findings indirectly.

### 4.2 Frequent invasion events of the populations concentrated in the Northern Hemisphere for over half a century

In the late 1950s, a parthenogenic populations of *L. oryzophilus* was first recorded in northern California (Lange and Grigarick, 1959). In the mid-1970s, the parthenogenetic populations of rice water weevils presumably “hitchhiked” across the Pacific into Aichi Prefecture, Japan (Iwata, 1979). After a decade of spreading, this populations quickly proliferated across the entire Japanese archipelago (Matsui, 1987). In Aichi Prefecture, Japan, the population is univoltine, with overwintered adults migrating to rice fields during daytime when temperatures exceed 20°C in May and June. By late July, eggs and larvae are found in the rice fields (Matsui, 1987). In southern Japan, the population is bivoltine, with very low larval densities on late-planted rice seedlings (Matsui, 1987). Despite agricultural and chemical control measures targeting the parthenogenetic populations in Japan, some individuals among the 150 rice varieties planted still exhibit high tolerance (Matsui, 1987). In 1988, the populations were recorded in mainland China and the Korean Peninsula, while Taiwan first reported their presence in Taoyuan County in March 1990 (Sun et al., 1996). In Korea, the populations were primarily univoltine, with occasional second-generation appearances in some years (Li, 1993; Sun et al., 1996). The spread and velocity of the populations in Korea are influenced by wind, mountainous terrain, and ground transportation (Uhm et al., 1989). Since the 21st century began, the populations were detected for the first time in Europe in 2004 (Caldara et al., 2004). Since then, the pest has rapidly spread across northern Italy, where rice cultivation is prevalent and the interconnected rice fields create corridors that aid its spread (Lupi et al., 2010; Wang et al., 2011). By 2020, this populations had established widespread populations in rice-growing regions across Europe, including Macedonia, Greece, France, Spain (Giantsis et al., 2017; Montauban et al., 2021).

### 4.3 The establishment hotspot areas concentrated in East Asia for the populations

The suitable areas of *L. oryzophilus* parthenogenetic populations were predominantly concentrated in rice-planting areas of the United States, Brazil, Argentina, Italy, Spain, Bulgaria, China, Nepal, Myanmar, Laos, Vietnam, India, the Korean Peninsula and Japan under current and future climate scenarios. It’s noteworthy that nearly all rice-growing areas in East Asia are covered by high-suitability areas for this populations, including China (northeastern, central and southern China), the Korean Peninsula, and Japan. Based on the results predicted by CLIMEX, Mao et al. (1997) found that the suitable areas are concentrated in Northeast, North, East, Central, South, and Southwest China. Qi et al. (2012) and Xie et al. (2022) used the MaxEnt model to analyze the suitable areas pattern of the populations in China reported high-suitability areas located primarily in northeastern, central and southern China. This also aligns with our results in China. Based on the analysis of climatic niche characteristics using the ecological niche hypervolume framework, the results indicate that compared to the native North American populations, the Asian populations occupied a larger climatic niche space, with significant climatic niche differentiation between the two populations. This isn’t surprising, as populations of *L. oryzophilus* that reproduce through parthenogenesis possess allowing them to rapidly adapt to changing environments and establish a sustainable populations within a short timeframe (Huang et al., 2017). Populations genetic studies have shown that there is a certain degree of genetic differentiation within the parthenogenetic populations between populations from the United States, Europe, and Asia, and among different geographic subgroups within Asia (Yang, 2008). Additionally, species invasion depends on the availability of host plants and the extent to which intruders require specific host plant species or are able to adapt to new species (Aljaryian et al., 2016). The extensive rice-growing areas in Asia have indeed provided a conducive environment for the spread of this populations to a certain extent (Huang et al., 2017). Furthermore, human activities have contributed to the spread of this beetle in Asia, especially in China. Despite geographical barriers, transporting rice seedlings, straw, and soil contaminated by the beetle could inadvertently introduce it to neighboring villages and counties (Huang et al., 2017). Notably, it appears that countries in West Africa, despite being major rice-growing regions, do not seem to face potential invasion by this populations. The two climatically distinct rice growing periods – dry and wet seasons – are crucial for rice double cropping in West Africa (Dingkuhn and Miezan, 1995). During the dry season, the region experiences low air temperatures and minimal solar radiation due to dry and dusty conditions. In the late dry season, temperature gradually temperatures gradually rise. During the wet season, the region shows a climate gradient from hot, humid and rainy (Average annual rainfall of 1150 mm) in the south to very hot and dry with low rainfall (Annual precipitation is about 200mm) in the north (Ibrahim et al., 2024). In Southeast Asia, the probability of extreme precipitation has increased from 1979 to 2019, with areas receiving over 2000 mm of annual rainfall accounting for 90.59% (Xu et al., 2022; Jin et al., 2023). This has contributed to a rise in flooding events, increasing the risk of waterlogging for the populations in this region (Aghaee and Godfrey, 2017). Therefore, extreme environmental changes have limited the occurrence of the rice weevil in West Africa to some extent. In the future, the establishment range of expansion will primarily be located in higher-altitude regions, such as the northeastern parts of Nepal and India. This is not surprising, as past studies have shown that global warming will further drive the beetles to expand into higher-altitude areas (Michael et al., 2022). This reflects the geographical response of insect to environmental changes, and it also indicates that the rice industry of these new regions will likely receive more attention in the future.

### 4.4 The potential economic loss of the populations is huge but recoverable in China

In recent decades, research on the potential economic impacts of IAPs on staple crop industry has significantly increased, focusing primarily on post-event impact assessments and evaluations of management effectiveness. For instance, Qin et al. (2020) utilized @RISK methodology to forecast the potential economic impact of *Spodoptera frugiperda* on maize industry in China. Without management measures, *S. frugiperda* is projected to cause potential economic losses ranging from 5.27 billion to 46.04 billion dollars (90% confidence level), while potential savings with management could amount to 3.57 billion to 40.93 billion dollars (90% confidence level).

In 1986, this species was classified as a national quarantine pest (Zhou, 1987). Two years later, a parthenogenetic population was first discovered in Tanghai, Hebei Province, and it rapidly spread along the coastal areas. Since the early 21st century, it has begun to move into central, southwestern, northwestern, and northeastern China (Huang et al., 2017). By July 2024, it had spread to 5/6 provinces in China (http://www.moa.gov.cn/govpublic/ZZYGLS/202409/t20240902_6461580.htm), becoming one of the most severe and rapidly spreading invasive pests in the country. Considering China is the largest producer of rice around the world, and the suitable areas of *L. oryzophilus* parthenogenetic populations cover nearly all rice-growing areas in China. Therefore, our study on potential economic losses focuses on China as a representative case. Based on @RISK software, the forecasted potential economic losses caused by *L. oryzophilus* parthenogenetic populations on rice industry show that the potential economic losses range from $5.44 billion to $32.46 billion USD (95% confidence level) under nil management scenario. However, the potential economic losses range from $0.10 billion to $0.83 billion USD (95% confidence level) after factoring in management costs, recovering over 90% of the potential economic losses. Sensitivity analysis reveals that the infestation rate of the populations and unit prevention cost is key input variable influencing potential economic losses under unmanaged scenario and managed scenario, respectively. Therefore, from an economic benefit perspective, implementing appropriate control measures in rice-growing regions affected by this global invasive pest is necessary and effective.

### 4.5 Better management of the establishment and spread of the populations

Since the initial discovery of *L. oryzophilus* parthenogenetic populations in the United States in 1958, the populations have rapidly spread across global rice-growing areas and establish stable populations. Our research highlights that East Asia contains extensive highly suitable areas for this populations, posing a significant threat to rice production in the region. Fortunately, a part of affected countries have implemented various control measures to mitigate the impact of the populations on local rice crops. In the U.S, as a semi-aquatic insect that relies on flooded conditions for larval development, the populations can be lower in a furrow irrigated rice system than the flooded field. Therefore, the furrow irrigated rice system may reduce the damage caused by this population (Kelly et al., 2021). During the fallow period, removing weeds from the rice field embankments and surrounding areas to reduce the abundance of host plants for the rice weevil may effectively lower its population density, thereby reducing the overall density in the rice fields (Palrang et al., 1994). This approach could be highly effective as it is easy for farmers to implement, making it suitable for promotion in China and other countries where the pest has become invasive (Huang et al., 2017). As a more efficient chemical control method, Chlorantraniliprole and its mixtures with other neonicotinoids chemicals (e.g., triazophos and thiamethoxam) are the primary chemical controls used in China (Huang et al., 2017). However, diamide seed treatments offer superior yield protection compared to neonicotinoids under high densities of the pest (Nicholas et al., 2024). Biological control and tolerance host plants breeding has shown applied promise. For instance, the highly resistant variety T03 was identified by comparing the adult feeding and oviposition preferences, egg hatching rate, larval survival rate among different rice varieties (Li et al., 2011). For potential but not yet realized suitable areas, it is crucial to establish official quarantine zones to prevent further spread. Strict quarantine on importing seedlings, straw, and soil from infected to non-infected areas, and avoiding the use of quarantine straw as packaging material, are essential (Huang et al., 2017).

## 5 Conclusion

Based on the analysis using the MaxEnt model, the precipitation of the warmest quarter (Bio18) has been identified as the primary environmental factor influencing the habitat suitability of *L. oryzophilus* parthenogenetic populations. During its invasion process, the populations has explored new climatic conditions, particularly in East Asia. The results indicate that this populations pose the greatest threat to major rice-producing regions in Asia under current and future climate scenarios, particularly rice-growing areas in China, the Korean Peninsula, and Japan. Specially, the populations would cost the rice industry in China 18.95 billion US dollars under unmanaged measures. Fortunately, climate change has not significantly altered the current global distribution pattern of this populations within the rice-growing regions and it is possible to recover 93% of the economic losses in China through reasonable chemical control, such as the application of triazophos and thiamethoxam. Our research findings will support in monitoring, surveillance, and the development of early warning systems to control *L. oryzophilus* parthenogenetic populations worldwide. Policymakers and governments can utilize these findings to develop effective integrated pest management strategies to handle potential future outbreaks of this populations.

## Data Availability

The raw data supporting the conclusions of this article will be made available by the authors, without undue reservation.
