# Supplementary material for: Assessing the establishment risk for parthenogenetic populations of Lissorhoptrus oryzophilus in global rice-growing areas and potential economic impact in China
Source: Front Plant Sci. 2025 Jan 22;15:1506418. doi: 10.3389/fpls.2024.1506418 (PMC11794189; doi:10.3389/fpls.2024.1506418)
Supplement: Supplementary file 1 [file DataSheet1.docx]

# Supplementary Material

Assessing the colonization risk of an invasive alien pest *Lissorhoptrus oryzophilus* parthenogenetic strain in global rice-growing areas and potential economic impact in China

**Table S1.** The basic data on the global invasion history of *Lissorhoptrus oryzophilus* (including countries, states, and provinces).

| Country | States / Provinces | First recording time | Reference |
| --- | --- | --- | --- |
| United States | California (CA) | 1958 | Newell, 1913 |
|  | Texas (TX) | 1904 | Jiang et al., 2008. |
|  | Louisiana (LA) | 1912 | Tucker, 1912 |
|  | Arkansas (AR) | 1909 | Isely et al., 1934. |
|  | Florida (FL) | 1916 | Blatchley et al., 1916. |
| China | Hebei (HB) | 1988 | Shang et al., 2004 |
|  | Tianjin (TJ) | 1990 |  |
|  | Beijing (BJ) | 1990 |  |
|  | Taiwan (TW) | 1990 |  |
|  | Liaoning (LN) | 1991 |  |
|  | Shandong (SD) | 1992 |  |
|  | Zhejiang (ZJ) | 1993 |  |
|  | Jilin (JL) | 1993 |  |
|  | Fujian (FJ) | 1996 |  |
|  | Hunan (HN), | 2001 |  |
|  | Anhui (AH) | 2001 |  |
|  | Shanxi (SX) | 2002 | Li et al., 2006 |
|  | Shaanxi (SN) | 2003 | Zhu et al., 2015 |
|  | Inner Mongolia Autonomous (NM) | 2005 |  |
|  | Yunnan (YN) | 2007 |  |
|  | Jiangxi (JX) | 2007 |  |
|  | Heilongjiang (HL) | 2007 |  |
|  | Hubei (HB) | 2008 |  |
|  | Guizhou (GZ) | 2010 | Zeng, 2010 |
|  | Xinjiang (XJ) | 2010 |  |
|  | Sichuan (SC) | 2013 | the National Agricultural Plant Quarantine Pests Distribution Administrative Region Directory（2012) |
|  | Guangxi (GX) | 2014 | the National Agricultural Plant Quarantine Pests Distribution Administrative Region Directory（2013) |
|  | Henan (HN) | 2015 | the National Agricultural Plant Quarantine Pests Distribution Administrative Region Directory（2014) |
|  | Ningxia (NX) | 2017 | the National Agricultural Plant Quarantine Pests Distribution Administrative Region Directory（2018) |
|  | Guangdong (GD) | 2018 | the National Agricultural Plant Quarantine Pests Distribution Administrative Region Directory（2018) |

| Country | States / Provinces | First recording time | Reference |
| --- | --- | --- | --- |
| Mexico | - | 1960 | Rodríguez et al., 1967 |
| Japan | - | 1976 | EPPO Global Database |
| Republic of Korea | - | 1980 | EPPO Global Database |
| Italy | - | 2003 | Caldara et al., 2004 |
| Greece | - | 2016 | Giantsis et al., 2017 |
| France | - | 2014 | EPPO Global Database |
| Spain | - | 2018 | EPPO Global Database |

**Table S2.** 19 bioclimatic factors from the World Climate Database

| Variable | Description | Unit |
| --- | --- | --- |
| Bio1 | Annual mean temperature | ℃ |
| Bio2 | Mean diurnal temperature range | ℃ |
| Bio3 | Isothermality | – |
| Bio4 | Temperature seasonality | – |
| Bio5 | Maximum temperature of warmest month | ℃ |
| Bio6 | Minimum temperature of coldest month | ℃ |
| Bio7 | Temperature annual range | ℃ |
| Bio8 | Mean temperature of wettest quarter | ℃ |
| Bio9 | Mean temperature of driest quarter | ℃ |
| Bio10 | Mean temperature of warmest quarter | ℃ |
| Bio11 | Mean temperature of coldest quarter | mm |
| Bio12 | Annual precipitation | mm |
| Bio13 | Precipitation of wettest month | mm |
| Bio14 | Precipitation of Driest Month | mm |
| Bio15 | Precipitation seasonality | – |
| Bio16 | Precipitation of Wettest Quarter | mm |
| Bio17 | Precipitation of warmest quarter | mm |
| Bio18 | Precipitation of Warmest Quarter | mm |
| Bio19 | Precipitation of coldest quarter | mm |

**Table S3.** Contribution rate of selected bioclimatic variables to the potential geographical distribution of *Lissorhoptrus oryzophilus* parthenogenetic populations.

| Variable | Description | Unit | Percent contribution (%) |
| --- | --- | --- | --- |
| Bio18 | Precipitation of Warmest Quarter | mm | 57.9 |
| Bio4 | Temperature seasonality | – | 22 |
| Bio10 | Mean temperature of warmest quarter | ℃ | 13.7 |
| Bio16 | Precipitation of Wettest Quarter | mm | 1.7 |
| Hii | Human influence index | – | 1.3 |
| Bio2 | Mean diurnal temperature range | ℃ | 1 |
| Bio15 | Precipitation seasonality | – | 0.9 |
| Bio19 | Precipitation of coldest quarter | mm | 0.8 |
| Alt | Altitude | m | 0.4 |
| Bio14 | Precipitation of Driest Month | mm | 0.4 |


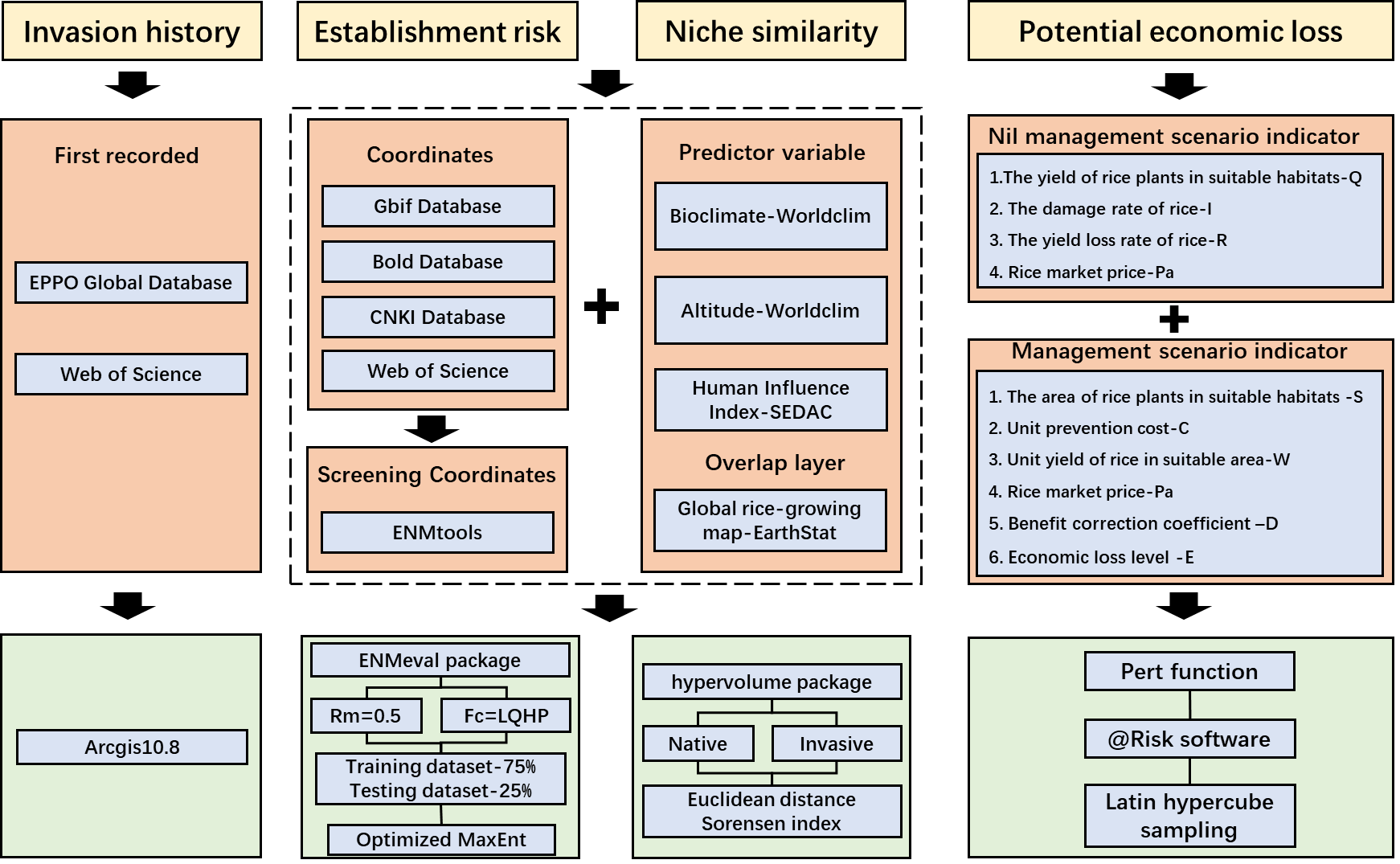


**Figure S1.** Research flow chart.


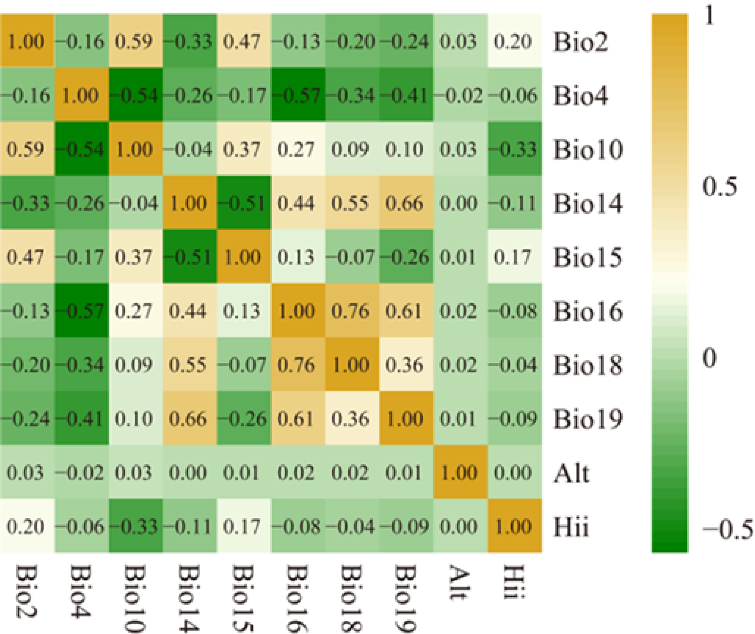


**Figure S2.** Correlation analysis of bioclimatic variables; ten environmental variables were retained (| *r* |< 0.8, Pearson).


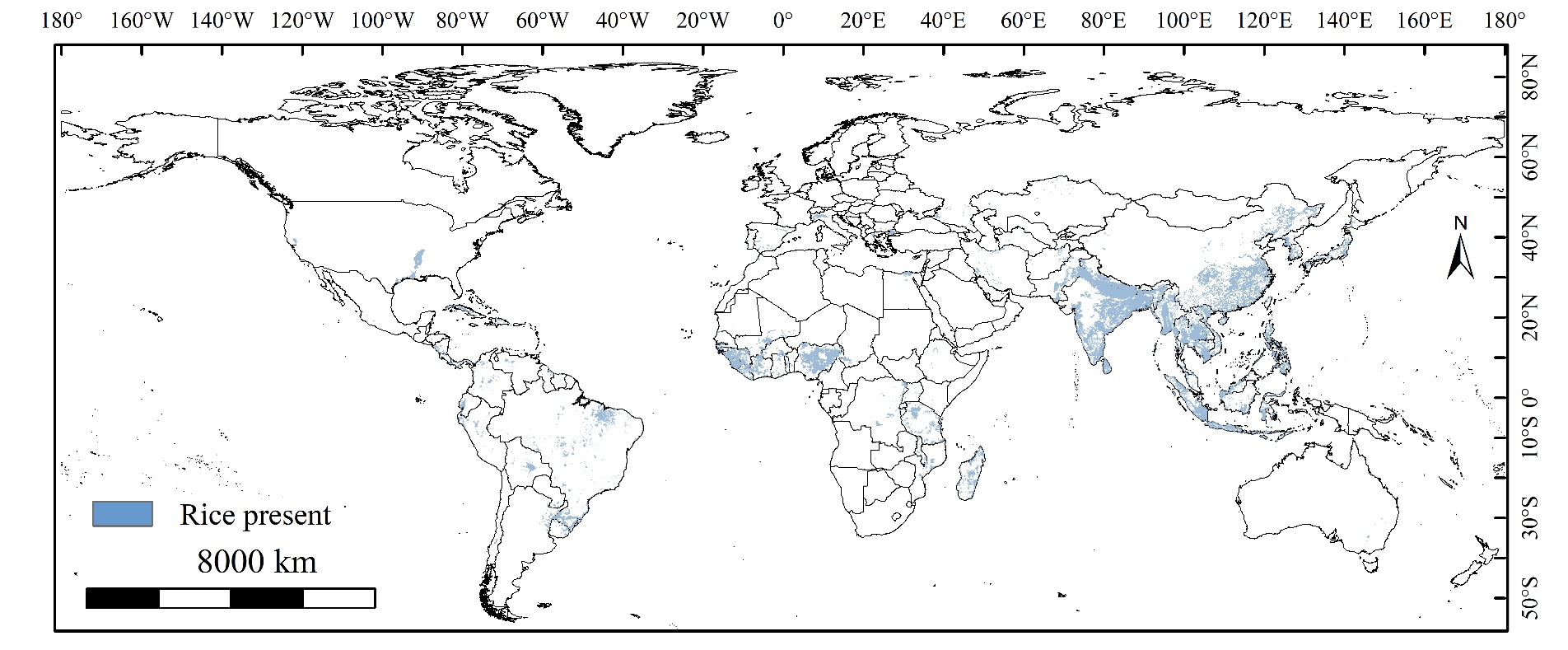


**Figure S3.** The distribution map of global rice-growing areas from the EarthStat database (www.earthstat.com).


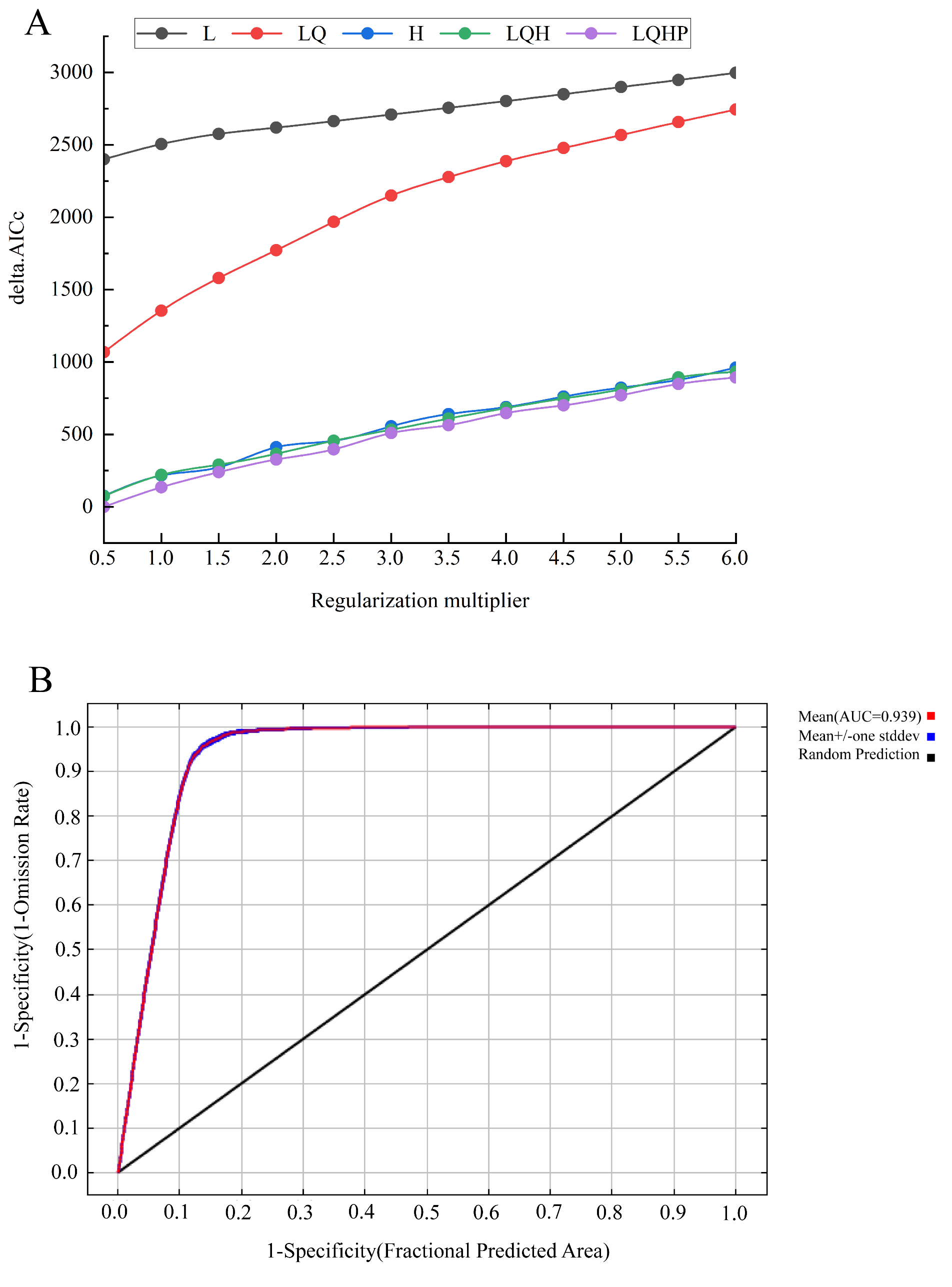


**Figure S4**. MaxENT model optimization and parameterization (A) AIC value of the parameter combination (FC, RM) based on ENMeval; (B) Parameter combination results for *Lissorhoptrus oryzophilus* parthenogenetic populations by ENMeval package.


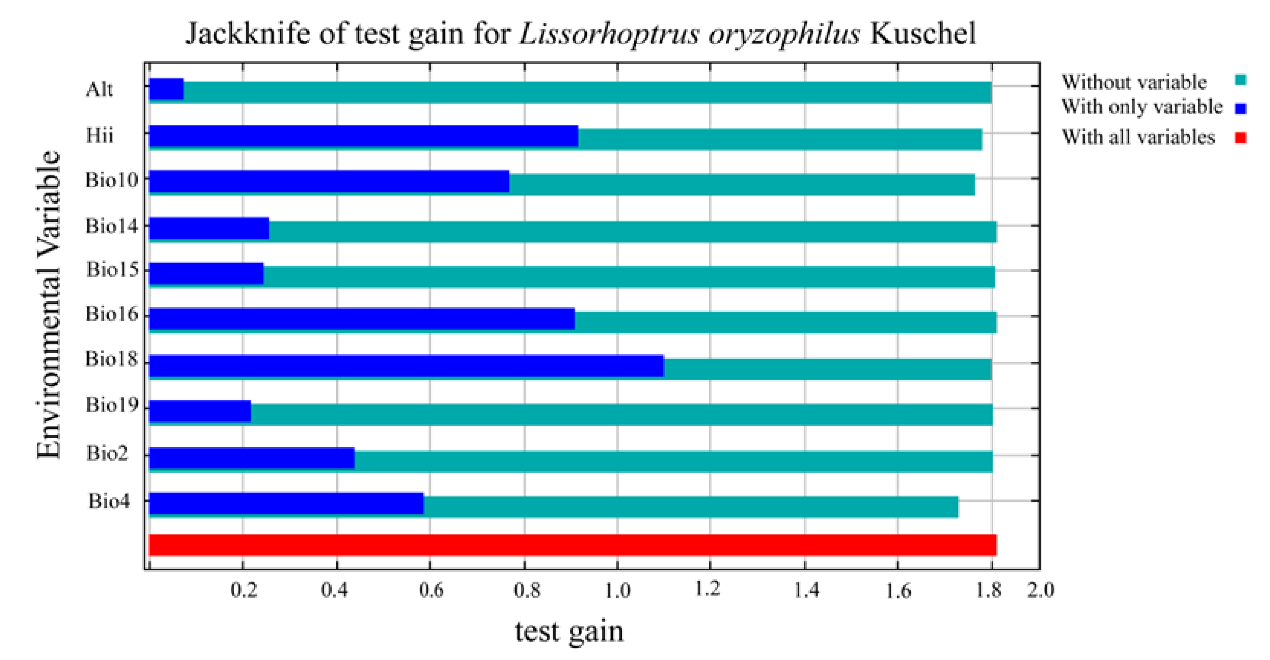


**Figure S5.** Jackknife results of relative importance of different predictor variables for *Lissorhoptrus oryzophilus* parthenogenetic populations.


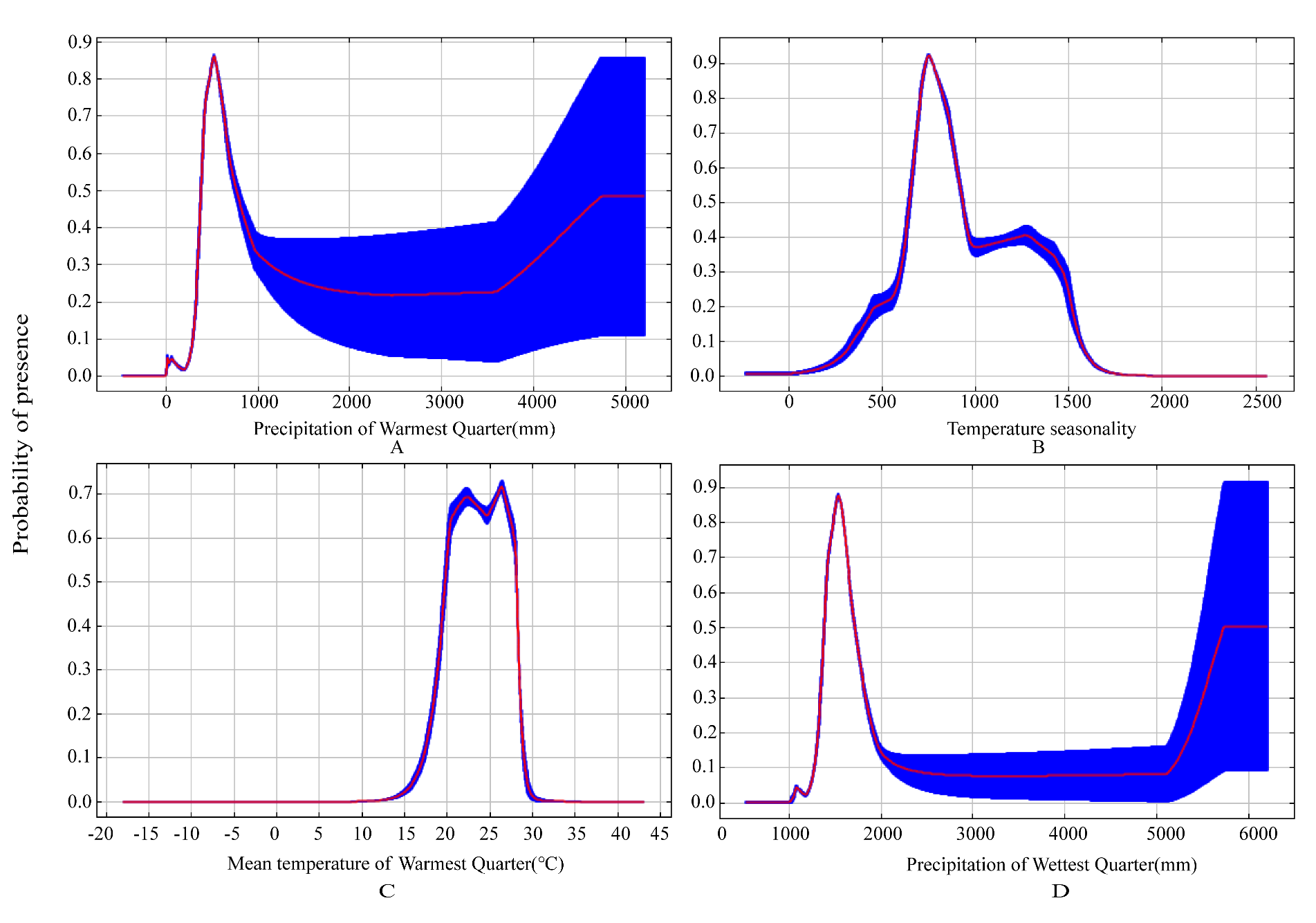


**Figure S6.** Response curves of the bioclimatic variables most related to the distribution of *Lissorhoptrus oryzophilus* parthenogenetic populations: (A) bio18 (Precipitation of Warmest Quarter); (B) bio4 (Temperature seasonality); (C) bio10 (Mean temperature of Warmest Quarter); (D) bio16 (Precipitation of Wettest Quarter).


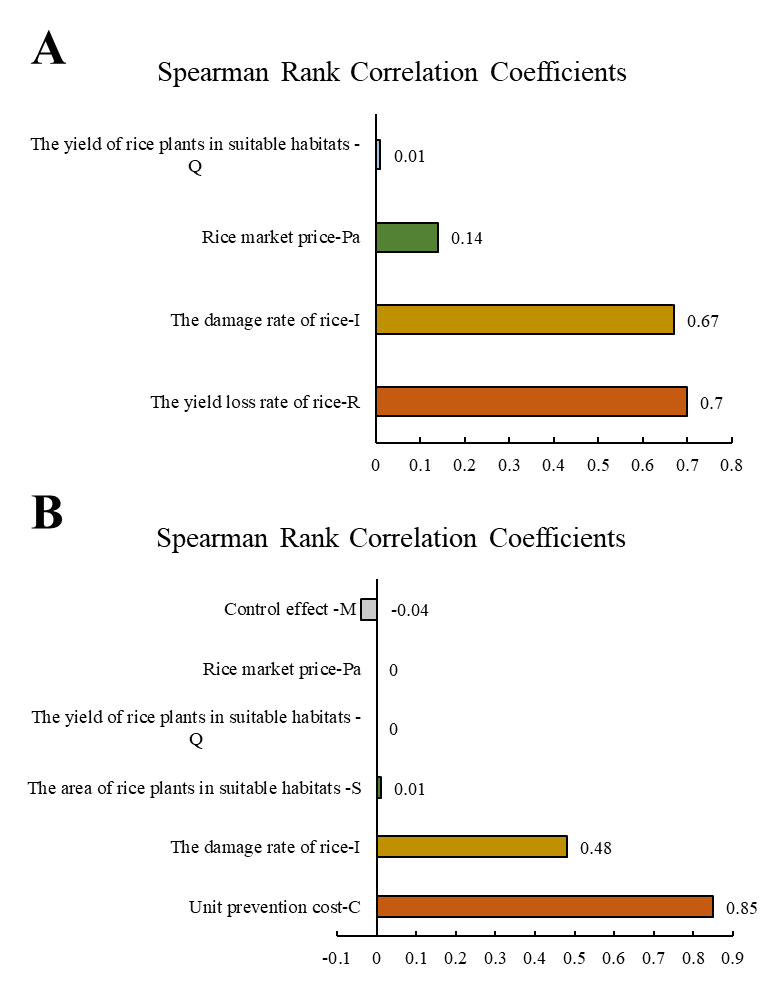


**Figure S7.** Sensitivity analysis results of potential economic loss of rice industry caused by *Lissorhoptrus oryzophilus* parthenogenetic populations under the unmanaged scenario (A) and the managed scenario (B) in China.

References

Newell, W. (1913). Notes on the biology of rice water weevil (Lissorhoptrus simplex Say) and its control. Journal of Economic Entomology, 6, 55–61. <https://doi.org/10.1093/jee/6.1.55>

Jiang, M., Way, M. O., Du, X., Ji, X., and He, Y. (2008). Reproductive biology of summer/fall populations of rice water weevil, Lissorhoptrus oryzophilus Kuschel, in southeastern Texas. Southwestern Entomologist, 33(2), 129–137. <https://doi.org/10.3958/0147-1724-33.2.129>

Tucker, E. S. (1912). The rice water-weevil and methods for its control. United States Department of Agriculture, No. 152, Washington, DC.

Isely, D., and Schwardt, H. H. (1934). The rice water weevil problem in Arkansas. Journal of Economic Entomology, 25(2), 218–222. <https://doi.org/10.1093/jee/25.2.218>.

Blatchley, W., and Leng, C. (1916). Rhynocophora or weevils of North Eastern America. Nature Publishing Co., Indianapolis, IN.

Shang, H., and Zhang, Z. (2004). Occurrence and control of *Lissorhoptrus oryzophilus* in China. In: Research on Contemporary Entomology - Proceedings of the 60th Anniversary of the Entomological Society of China, (Zhejiang University of Science and Technology; China National Rice Research Institute), p. 5.

Rodríguez, R. R., and Sifuentes, A. A. (1967). The rice borer in the state of Morelos. Circular CIB 12, SAG, INIA, CEZACA.

Caldara, R., Diotti, L., & Regalin, R. (2004). First record for Europe of the rice water weevil, *Lissorhoptrus oryzophilus* Kuschel Coleoptera, Curculionoidea, Erirhinidae Prima segnalazione per lEuropa di *Lissorhoptrus oryzophilus* Kuschel Coleoptera, Curculionoidea, Erirhinidae, temibile parassitadi Oryza sativa L. Bollettino di Zoologia Agraria e di Bachicoltura, 36(1), 165-171.

Giantsis, I. A., Castells Sierra, J., and Chaskopoulou, A. (2017). The distribution of the invasive pest, rice water weevil Lissorhoptrus oryzophilus Kuschel (Coleoptera: Curculionidae), is expanding in Europe: First record in the Balkans, confirmed by CO1 DNA barcoding. Phytoparasitica, 45, 147–149. <https://doi.org/10.1007/s12600-017-0576-z>

Shang, H., and Zhang, Z. (2004). Occurrence and control of *Lissorhoptrus oryzophilus* in China. Research on Contemporary Entomology, p. 5 (In Chinese).

Li, J., Zheng, W., Yin, L., et al. (2006). Occurrence, damage, and integrated control measures of *Lissorhoptrus oryzophilus* in Shanxi Province. Plant Protection Guide of China, (12), 16–17 (In Chinese).

Zhu, X., Luo, J., and Tian, Z. (2015). Research progress on *Lissorhoptrus oryzophilus* in China. Green Prevention and Control of Pests and Diseases and Agricultural Product Quality Safety, p. 2 (In Chinese).

Zeng, J. (2010). First occurrence of the invasive species Lissorhoptrus oryzophilus in Guizhou Province. China Meteorological News, p. 3 (In Chinese).
